# Supplementary material for: De novo design of high-affinity binders of bioactive helical peptides
Source: Nature. 2023 Dec 18;626(7998):435–42. doi: 10.1038/s41586-023-06953-1 (PMC10849960; doi:10.1038/s41586-023-06953-1)
Supplement: Supplementary file 1 — Supplementary Methods with additional references, Tables 1–9 and Figs. 1–12. [file 41586_2023_6953_MOESM1_ESM.pdf]

nature portfolio

https://doi.org/10.1038/s41586-023-06953-1

Supplementary information

De novo design of high-affinity binders of bioactive helical peptides

In the format provided by the authors and unedited

Supplementary Methods for De novo design of high-affinity protein binders to bioactive helical peptides

Experimental Methods

Gene construction of peptide hormone binders

The designed protein sequences were optimised for expression in *E. coli*. Linear DNA fragments (eBlocks, Integrated DNA Technologies) encoding design sequences included overhangs suitable for cloning into pETcon3 vector for yeast display[7] (deposited in Addgene with the number #45121) and Golden Gate cloning into LM627 vector for protein expression (Addgene #191551)[31]. For initial testing hallucinated binders to Bid, binders were cloned into a modified LM627 vector. Specifically, Golden Gate cloning was used to generate sfGFP-Bid-STOP-[Binder]-SNAC-HISx6 assemblies.

Yeast display screening

For the yeast transformation, 50-60 ng of digested pETcon3 with the NdeI and XhoI restriction enzymes and 100 ng of insert (eBlocks, Integrated DNA Technologies) were transformed into *S. cerevisiae* EBY100 strain using the protocol described in ref[7]. EBY100 cultures were grown in C-Trp-Ura medium supplemented with 2% (w/v) glucose (CTUG). For induction of expression, yeast cells initially grown in CTUG were transferred to SGCAA medium supplemented with 0.2% (w/v) glucose and induced at 30 °C for 16–24 h. Cells were washed with PBSF (PBS with 1% (w/v) BSA) and labelled for 40 minutes with biotinylated peptide targets at room temperature using without-avidity labelling conditions[7]. After incubation time, cells were washed and resuspended in PBSF for individual sorting of cells harbouring each unique design using a 96-well compatible autosampler in the Attune NxT Flow Cytometer (Thermo Fisher Scientific).

NanoBiT screening

Linear gene fragments encoding binder design sequences and target peptide sequences were cloned into *E. coli* expression vectors using Golden Gate assembly; these vectors were pET28b(+) derivatives genetically fusing the smBiT and lgBiT halves of the NanoLuc® Luciferase (Promega) to the binders and peptides respectively. Resulting plasmids were transformed into BL21* (DE3) (Invitrogen) *E. coli* competent cells, then grown in 1mL TBII in 96-deepwell plates at 37°C and 600 rpm. After 2 hours, expression was induced with IPTG (0.1 mM) and cells were incubated for an additional 4 hours. Cells were harvested by centrifugation (15 min at 4000 x g), then resuspended in 100 µL lysis buffer (10 mM NaP pH 7.4, 150 mM NaCl, 5 mM $MgCl_2$, 1 mg/mL lysozyme, 10 µg/mL DNAse I, 1 tablet Complete Protease inhibitor / 50 mL). Cells were incubated for 1 hour at room temperature and 600 rpm, then frozen (-80C for 30min) and thawed (37°C at 600 rpm for 30min) twice. Lysate was cleared by centrifugation (20 min at 4000 x g), and the soluble fraction was then transferred to a 96-well plate for use as stock protein/peptide for conducting the nanoBiT screen. Screens were assembled in 96-well Half Area Black Flat Bottom Polystyrene NBS Microplates (Corning 3686). Binder design smBiT lysate was diluted 12 µL into 1400 µL assay buffer (10 mM NaP pH 7.4, 150 mM NaCl), while

target peptide lgBiT lysate was diluted 6 μL into 1400 μL assay buffer. Stock rows in the assay plate were prepared by mixing 40 μL substrate (499.2 μL assay buffer, 20.8 μL Nano-Glo® Luciferase Assay Substrate (Promega)) with 40 μL diluted binder design smBiT lysate, while experimental rows were prepared by adding 50 μL diluted target peptide lgBiT lysate. At read time, 50 μL of the stock row was added to the 50 μL experimental row and mixed quickly and carefully, then luminescence was read immediately for 5 min using a plate reader (Biotek Synergy Neo2).

Identification of weak binder hits from parametric designs

The first helical peptide binder hits were identified in experiments screening for binding using the nanoBiT split luciferase assay. These kinetic binding experiments were performed in cell lysate with no control over protein concentration, so candidate binders were selected qualitatively for showing some increase in luminescence signal over time above background noise, indicating likely binding activity. Additional binding curve experiments (Supplementary Fig. S4) indicated that this binding activity was at very weak affinities, likely >100 nM (indistinguishable from the background signal of weak luciferase binding for the assay). Therefore, these initial candidates were not further characterised, but rather selected for additional design to yield higher affinity binders.

Bicistronic protein expression

Hallucinated binders to Bid were screened by bicistronic expression with the Bid peptide. Plasmids encoding sfGFP-Bid-STOP-[Binder]-SNAC-HISx6 were cloned into *E. coli,* and 2 mL cultures of each of the 47 designs were grown overnight in LB. Cultures were diluted into TB medium, and grown to approximately OD280 0.6, before induction with 1 mM IPTG for 4 hours at 37°C. Bacteria were lysed for 15 minutes in 300 B-PER (Thermo) + 1 mM PMSF, 0.1 mg/mL Lysozyme (Sigma), 0.01 mg/mL DNAse I. Lysates were clarified by centrifugation at 4000 *g* for 10 minutes, before purification on Ni-NTA resin (wash buffer: 20 mM Tris pH 8.0, 150 mM NaCl, 20 mM Imidazole; elution buffer: 20 mM Tris pH 8.0, 150 mM NaCl, 250 mM Imidazole). Eluates were assessed for GFP fluorescence on a fluorescence plate reader.

Peptide synthesis and purification

The PTH-TAMRA peptide was synthesised in-house on a CEM Liberty Blue microwave synthesiser. All L- and D-amino acids were purchased from P3 Biosystems. Oxyma Pure was purchased from CEM, DIC was purchased from Oakwood Chemical, diisopropyl ethylamine (DIEA) and piperidine were purchased from Sigma- Aldrich. Dimethylformamide (DMF) was purchased from Fisher Scientific and treated with an Aldraamine trapping pack prior to use. Synthesis was done on a 0.1 mmol scale on CEM Cl-TCP(Cl) resin. Five equivalents of each amino acid were activated using 0.1 M Oxyma with 2% (v/v) DIEA in DMF, 15.4% (v/v) DIC, and coupled on resin for 4 min with double coupling if needed. This was followed by deprotection using 5 mL of 20% piperidine in DMF for 2 min at 95 °C. Global deprotection was accomplished TFA/Water/TIPS (95:2.5:2.5) for 3 hours. This deprotection mixture was precipitated in 30 mL of ice-cold ethyl ether, centrifuged and decanted, then washed twice more with fresh ether and

dried under nitrogen to yield crude peptide for high pressure liquid chromatography (HPLC) purification.

The TAMRA-Bid peptide was synthesized using a PurePep Chorus peptide synthesizer (Gyros Protein Technologies). Fmoc-protected amino acids were purchased from Ambeed. The peptide synthesis was performed at a 0.05 mmol scale on Rink amide AM resin (Matrix Innovation). Following reagents were prepared in dimethylformamide (DMF, VWR) for amino acid coupling: Fmoc-protected amino acids (0.2 M), O-(1H-6-Chlorobenzotriazole-1-yl)-1,1,3,3-tetramethyluronium hexafluorophosphate (HCTU, 0.5 M, Ambeed), and diisopropyl ethylamine (DIEA, 1.0 M, Thermo Scientific). For peptide elongation, the resin was washed with DMF (3x 3 mL) before fmoc-deprotection was carried out using 3 mL 20 % (v/v) piperidine (Sigma-Aldrich) in DMF (2x, 2 min, room temperature, agitating at 300 rpm). After a DMF washing step (3x 3 mL), amino acid/HCTU/DIEA (1:1.25:2.5) was added to the resin in 5-fold excess and agitated (2x 10 min, 50 °C). After a final fmoc-deprotection, N-terminal coupling of 5-(and-6)-carboxytetramethylrhodamine (TAMRA, Anaspec Inc.) was accomplished using following conditions: TAMRA:benzotriazol-1-yloxy)tripyrrolidinophosphonium hexafluorophosphate (PyBOP, Novabiochem):DIEA (1.5:1.5:3) in N-Methyl-2-pyrrolidone (NMP, Sigma-Aldrich) for 16 h at room temperature and 300 rpm. Tin foil was used to cover the reaction vessel to avoid photobleaching of the fluorophore. For global deprotection and cleavage from the resin, the peptide was incubated with TFA/Water/triisopropyl silane (TIPS, Sigma-Aldrich)/ 1,2-Bis(2-mercaptoethoxy)ethane, 3,6-Dioxa-1,8-octane-dithiol (DODT, Sigma-Aldrich) (92.5:2.5:2.5:2.5) for 2 h at room temperature while agitating. 30 mL of ice-cold diethyl ether (VWR) was added for peptide precipitation and the precipitated peptide was centrifuged at 2500 x g for 10 min at 4 °C. The supernatant was discarded, and the peptide was washed with 20 mL of diethyl ether. The peptide pellet was dissolved in water/acetonitrile/TFA (50:50:0.1) and lyophilized.

The crude peptide was dried and dissolved in a mixture of ACN and water where the entire crude is soluble. This solution was purified on a C18 column in an Agilent HPLC instrument. A linear gradient of increasing ACN with 0.1% TFA was used to purify the samples. UV signal was monitored at 214 nm and all peaks were collected. Peaks were checked using ESI mass spectroscopy for the correct peptide mass. The purified peptide was then lyophilized for further use.

Protein expression and purification in *E. coli* for peptide hormone binders

Protein expression was performed using 50 mL of the Studier autoinduction media supplemented with kanamycin, and grown overnight at 37°C. The cells were harvested by spinning at 4,000 x g for 10 min and then resuspended in lysis buffer (100 mM Tris-HCl, 200 mM NaCl, 50 mM imidazole) supplemented with protease inhibitor tablets (Pierce™ Protease Inhibitor Tablets, EDTA-free). Then, the cells were lysed by sonication in a Qsonica, Q500 with a: 4-pronged horn for 2:30 min ON total, with an amplitude of 80%. Soluble fractions were clarified by centrifugation at 14,000 x g for 40 minutes, and were subsequently purified by affinity chromatography using bed Ni-NTA resin (Qiagen or Thermo Fisher) on a vacuum

manifold. A series of washes using Low-salt buffer (20 mM Tris-HCl, 200 mM NaCl, 50 mM imidazole) and High- salt buffer (20 mM Tris-HCl, 1000 mM NaCl, 50 mM imidazole) were performed prior to elution with Elution buffer (20 mM Tris-HCl, 200 mM NaCl, 500 mM imidazole). After elution, protein samples were filtered and injected into an autosampler-equipped Akta pure system on a Superdex S75 Increase 10/300 GL column at room temperature. The SEC running buffer was 20mM Tris-HCl, 100mM NaCl pH 8. We pooled the largest abundance monodisperse peak fractions and concentrated using Spin filters (3 kDa molecular weight cutoff, Amicon, Millipore Sigma) and stored at 4 °C before downstream characterizations. Protein concentrations were determined by absorbance at 280 nm using a NanoDrop spectrophotometer (Thermo Scientific) using their extinction coefficients and molecular weights obtained from their amino acid sequences using the ProtParam tool. We additionally verified the monodispersity of the pooled fractions by reinjecting them on the same column for the majority of the binders after 24h (Supplementary Fig. S18)

Fluorescence polarisation

Fluorescence polarisation binding assays were carried out in 96-well plates (Corning 3686), with two-fold serial dilution of designed peptide binders in the presence of 0.5 nM fluorescently labelled peptide targets. Protein and peptide were diluted from their stock concentration into 20mM Tris-HCl pH 8, 100mM NaCl, 0.1% v/v Tween 20, and the protein was titrated in 2-fold serial dilutions onto constant peptide. After incubating the peptide and binder for one hour at room temperature, the fluorescence polarisation was measured at the excitation and emission wavelengths of the FAM dye (485/530 nm) or the TAMRA dye (530/590 nm), in a Synergy Neo2 multi-mode plate reader. Titrations were conducted in replicate, and the K_d was fitted with SciPy[59]. Specifically, curves were fit to N observations of an observed signal, $Signal_i$, at titrated concentrations $[A_{tot}]_i$ according to the following equation:

$$Signal_i = Baseline + Amplitude\,\frac{AB_{conc}([A_{tot}]_i, [B_{tot}], K_d)}{[B_{tot}]},$$

Where $[B_{tot}]$ is the known total concentration of the binder, $Baseline$ and $Amplitude$ are free parameters, and the concentration of the bound state $[AB]$ is computed as

$$AB_{conc}([A_{tot}]_i, [B_{tot}], K_d) = (([A_{tot}] + [B_{tot}] + K_d) \pm \sqrt{([A_{tot}] + [B_{tot}] + K_d)^2 - 4[A_{tot}][B_{tot}]})/2$$

The unknown parameters (K_D, $Baseline$ and $Amplitude$) were fit using scipy.optimize.curve_fit, $[B_{tot}]$was additionally fit in the optimization, but only allowed to within 0.5 nM $\pm\ 0.1\%$.

Fluorescence polarisation measurements for TAMRA-Bid were performed in 96-well, flat-bottom, half-area microplates (Corning 3881) using a CLARIOstar Plus plate reader (BMG Labtech) set to excitation and emission wavelengths of 540 and 590 nm, respectively. TAMRA-Bid peptide and hallucinated proteins were diluted into a buffer containing 50 mM sodium phosphate pH 7.0 and 0.05 % v/v Tween-20. The concentration of TAMRA-Bid peptide was kept constant at 10 nM. For the proteins, a 2-fold serial dilution was performed and added to the peptide. After one to four hours of incubation at room temperature, FP measurements were conducted. Titrations were carried out in triplicates.

Peptides used for the assay are shown in Supplementary Table 1

Supplementary Table 1. Fluorophore-labelled peptides used in Fluorescence polarisation assays

Peptide name	Sequence	Supplier	Cat #	Fluorophore
PTH-TAMRA	SVSEIQLMHNLGKHLNSMERVE WLRKKLQDVHNF	In-house	NA	5-TAMRA
PTHrp-FAM	AVSEHQLLHDKGKSIQDLRRRF FLHHLIAEIHTAEIA	Phoenix Pharmaceu ticals, Inc.	FG-056- 08A	FAM
SCT-FAM	HSDGTFTSELSRLREGARLQRL LQGLV	Phoenix Pharmaceu ticals, Inc.	FG-067- 03A	FAM
GCG-FAM	HSQGTFTSDYSKYLDSRRAQD FVQWLMNT	Addex Bio	ABBFO 2033	FAM
NPY-FAM	SKPDNPGEDAPAEDMARYYSA LRHYINLITRQR	Phoenix Pharmaceu ticals, Inc.	FG-049- 04A	FAM
PPY-FAM	IKPEAAGEDASPEELNRYYASL RHYLNLVTRQRY	Phoenix Pharmaceu ticals, Inc.	FG-059- 02A	FAM

TAMRA-Bid	TAMRA-(b-A)-QEDIIRNIARHLA QVGDSMDR SIPPG	In-house	NA	5-TAMRA

Cloning, expression and purification of Bid-binding hallucinations, Avi-tagged Bid peptide and MCL-1

Bid-binding Hallucinations were cloned into a pET28 vector, containing an N-terminal His_{10} and a PreScission cleavage site, using TEDA cloning[60] and transformed into XL-1-Blue chemically competent cells, single clones isolated and amplified and sequences confirmed by Sanger sequencing. Plasmids transformed into chemically competent BL21 DE3 *E. coli*, and plated onto LB agar plates supplemented with 100 µg/mL kanamycin. Single colonies were used to make starter cultures of LB with 100 µg/mL kanamycin and incubated overnight at 37 °C. 1:100 volume starter culture was added to autoinduction media Overnight Express Instant TB Medium (Novagen) in Ultra-Yield flasks (Thomson), with 100 µg/mL kanamycin, incubated at 37 °C for 5 hours, then 18 °C for 18 hrs. Cells harvested by centrifugation 6,000 rpm, 20 mins, 4 °C, and pellets were frozen at -80°C.

Defrosted cell pellets were resuspended in approx. 10 mL/g Lysis Buffer (50 mM potassium phosphate pH 7.0, 300 mM NaCl, 5 mM imidazole, 2 mM b-mercaptoethanol, 10% glycerol), supplemented with 60 µg/mL lysozyme, 1.4 µg/mL DNaseI, 0.05 mM PMSF. Cells were lysed by passing through the French press twice, 18 kpsi. Lysate was clarified by centrifugation 18,000 x g, 45 mins, 4 °C, and loaded onto HIS-Select Nickel affinity resin (Sigma) by gravity, resin washed with Wash Buffer (50 mM potassium phosphate pH 7.0, 100 mM NaCl, 5 mM imidazole, 2 mM b-mercaptoethanol, 10% glycerol) and eluted with Wash Buffer containing 350 mM imidazole. Protein containing fractions (assessed by A_{280}) were combined, and further purified by size exclusion chromatography (SEC) using HiLoad 16/600 200 pg Superdex column (Cytiva) using ÅKTA FPLC system (Cytiva) equilibrated in 50 mM sodium phosphate pH 7.0, 1 mM DTT. Fractions were concentrated, concentration measured using A_{280} and predicted extinction coefficients[33], then flash frozen $N_{2(l)}$ for storage at -80 °C.

DNA corresponding to BH3 motif of human Bid Q79-G144 (Uniprot: P55957) was assembled by complementary oligos (IDT) and primer extension using Klenow fragment (NEB), and cloned using TEDA into pET28 with an N-terminal His_{10}, SUMO and C-terminal Avi. Expression and purification was carried out as for the Hallucinations, except for co-transformation with a chloramphenicol-resistant BirA expressing plasmid, the addition of chloramphenicol 25 µg/mL in all cultures, with the addition of 40 µM BTN to the media before temperature was reduced to 18 °C. After SEC, His_{10}-SUMO was cleaved using ULP-1 protease, and His_{10}-SUMO removed using Ni resin, Bid-Avi peptide concentration was measured using A_{280}, and stored at -80°C. To express human Mcl-1 P166-G327 (Uniprot: Q07820) a pEQ80L vector with N-terminal His_6 and Avi-tag, for co-expression with BirA. Expression and purification was carried out as for the Bid-binding hallucinations, with the addition of 40 µM BTN to the media before temperature was reduced to 18 °C.

ITC

Isothermal titration calorimetry was carried out with an ITC200 (Micocal). Bid peptide was in the syringe, at ~300 μM, and binder (Hallucination of Mcl-1) was kept in the cell (~25 μM), with both peptide and binder in matched buffer (sodium phosphate pH 7.0, 1 mM DTT). Temperature was held at 25 °C or 10 °C, as indicated. Fitting of titrations was carried out using 1-site binding, using manufacturers software (OriginLab).

Circular dichroism

Spectra were recorded for Bid peptide alone, Bid in complex with binders (Hallucination or Mcl-1) and binders alone. All concentrations were 10 μM, in a 2 cm pathlength quartz cuvette. Spectra recorded on J-1500 Circular Dichroism Spectrophotometer, with temperature held at 25 °C, or ramped at 1 °C/min.

Pull-down

10 μL bead slurry Dynabeads M-280 Streptavidin (Thermo Fisher Scientific) were washed with Pull-Down Buffer (sodium phosphate pH 7.0, 1 mM DTT, 0.05% Tween20), incubated with saturating amounts of (Avi-tagged) Bid peptide 15 mins, 4 °C with rotation, beads were then incubated with free biotin 25 μM, and washed three times with ice cold Pull-Down Buffer. 10 μL of 2 μM binder (hallucination or Mcl-1) was incubated with pelleted beads for 30 mins, 4 °C, with rotation. Supernatant was recovered and the beads washed three times before resuspension in 10 μL Pull-Down Buffer. Both supernatant and washed beads were loaded onto denaturing SDS-PAGE, with protein detection by InstantBlue Coomassie staining.

Crystallisation and Structure Determination

All crystallisation experiments were conducted using the sitting drop vapour diffusion method. Crystallisation trials were set up in 200 nL drops using the 96-well plate format at 20 ˚C. Crystallisation plates were set up using a Mosquito LCP from SPT Labtech, then imaged using UVEX microscopes and UVEX PS-256 from JAN Scientific. Diffraction quality crystals formed in 0.2 M Ammonium chloride 0.1 M Tris pH 8 20% (w/v) PEG 6000 for GCG_partdiff; in 0.9 M Halogens, 0.1 M Tris- Bicine pH 8.5 Buffer, and 37.5% of 25% v/v MPD; 25% PEG 1000; 25% w/v PEG 3350 mixture for GCG_inpaint. In 0.1 M Citric acid pH 2.5, 20% (w/v) PEG 6000. PHT 0.2 M Sodium chloride for Bim_fulldiff, and in 0.1 M Sodium acetate pH 4.5, and 1.26 M Ammonium sulphate for PTH peptide only.

Diffraction data was collected at the Advanced Photon Source (APS) beamline 24-ID-C. X-ray intensities and data reduction were evaluated and integrated using XDS[61] and merged/scaled using Pointless/Aimless in the CCP4 program suite[62]. Starting phases were obtained by molecular replacement using Phaser[63] using the designed model for the structures. Following molecular replacement, the models were improved using phenix.autobuild[64]; efforts were made to reduce model bias by setting rebuild-in-place to false, and using simulated annealing and prime-and-switch phasing. Structures were refined in Phenix[64]. Model building was performed

using COOT[65]. The final model was evaluated using MolProbity[66]. Data collection and refinement statistics are recorded in Supplementary Table 9. Data deposition, atomic coordinates, and structure factors reported in this paper have been deposited in the Protein Data Bank (PDB), http://www.rcsb.org/ with accession code 8GJI (GCG_partdiff), 8GJG (GCG_inpaint), 8T5E (Bim_fulldiff), and 8T5F (PTH peptide only).

Design and characterization of lucCagePTH biosensor for parathyroid hormone detection

The detailed design protocol for the lucCage and lucKey sensor system was described previously[39]. In brief, the amino acid sequence (FELLDKLIELLRELIELTREYI) at the N-terminal end of the 6.1 nM PTH binder was grafted onto the latch region (residues 323 to 353) of lucCage. The Rosetta models were visually inspected and eight of them were selected for experimental validation. We produced, purified, and screened for the luminescence signal emitted from each biosensor in the presence of 5 µM PTH. From this process, we identified several hits showing increased luminescence upon adding PTH, of which we assigned the best one with a 21-fold activation as lucCagePTH. We then set up assays to evaluate the response of lucCagePTH with a range of PTH concentrations. 10 µl of 10 nM lucCagePTH, 10 µl of 10 nM lucKey, 10 µl of serial diluted PTH, and 40 µl of buffer (50% HBS-EP/50% Nano-Glo luciferase assay buffer) were pre-mixed and 30 µl of 100× diluted furimazine was injected immediately before luminescence kinetic acquisition. The luminescence measurements were taken every 1 min (0.1 s integration and 10 s shaking during intervals) for a total of 60 mins by Neo2 microplate reader. The linear region of luminescence responses to the corresponding PTH concentrations was fitted to a linear regression curve and the LOD was calculated as 3 × standard deviation of the response / the slope of the calibration curve.

Affinity enrichment of PTH analysed by LC-MS/MS

PTH-minibinder Bead preparation

PTH binder-conjugated beads were prepared by binding 1-10 mg of cysteine-containing protein binder at the C-terminus to SulfoLink™ Coupling Resin (ThermoFisher). Following the binding step, the excess active groups on the beads were blocked by adding 1 mL of 50 mM L-Cysteine•HCl and incubating for 30 minutes.

Sample description

Recombinant human PTH protein was purchased from Sigma (#SAE 0192_100 µg, MA, USA) and reconstituted at 100 µg/mL in a 10 % acetonitrile, 0.1 % formic acid,1 mg/mL bovine serum albumin solution and stored in 40 µL aliquots at -20 °C. Dilutions at 1000 ng/mL and 62.5 ng/mL were prepared freshly as needed by dilution in the same acetonitrile, formic acid, albumin solution.

The plasma samples used were de-identified clinical samples obtained from the clinical laboratories at the University of Washington Medical Center. The use of de-identified leftover

clinical samples was reviewed by the University of Washington Human Subjects Division (STUDY00013706).

The evaluation of PTH immunoaffinity enrichment in buffer and plasma was performed in three process replicates using 8 different types of samples:

- Series A: Reconstitution buffer (10 % acetonitrile, 0.1 % formic acid,1 mg/mL bovine serum albumin in water) served as the blank.
- Series B: Reconstitution buffer spiked with PTH at 7.2 ng/mL was directly digested without the addition of beads and served as the Control sample (representing 100% recovery of PTH).
- Series C: Reconstitution buffer spiked with PTH at 7.2 ng/mL was incubated with beads blocked by bovine serum albumin before washing and digestion, which served as the negative control, to quantify non-specific binding in buffer.
- Series D: Reconstitution buffer spiked with PTH at 7.2 ng/mL was incubated with designed binder-conjugated beads before washing and digestion, which was used to quantify the affinity precipitation of PTH from buffer.
- Series E: Plasma was incubated with beads blocked by bovine serum albumin before washing and digestion, which was used to quantify non-specific binding in unspiked plasma.
- Series F: Plasma was incubated with designed binder-conjugated beads before washing and digestion, which was used to quantify affinity precipitation of PTH in plasma.
- Series G: Plasma spiked with PTH at 7.2 ng/mL was incubated with beads blocked by bovine serum albumin before washing and digestion, which was used to quantify non-specific binding in spiked plasma.
- Series H: Plasma spiked with PTH at 7.2 ng/mL was incubated with designed binder-conjugated beads before washing and digestion, which was used to quantify the affinity precipitation of PTH in spiked plasma.

Sample preparation and LC-MS/MS conditions

Affinity enrichment was performed in buffer or plasma at the protein level. Designed binders were conjugated to tosyl-activated Dynabeads M-280 according to the manufacturer's instructions and subsequently blocked using bovine serum albumin and Tris. The amino terminal peptide was analysed after tryptic digestion of either pure protein in buffer, or after trypsin digestion of PTH that had been affinity precipitated by the designed binder-conjugated beads (or by the control/blocked magnetic beads). Briefly, PTH proteins in buffer/plasma were purified using PTH mini-binder conjugated-paramagnetic beads at room temperature, for 1 h. The beads were then washed 4 times with phosphate-buffered saline supplemented with CHAPS (0.1% 3-((3cholomidopropyl) dimethylammonio)-1-propanesulfate to reduce nonspecific interactions). The proteins that were affinity precipitated by the designed binder-conjugated-paramagnetic beads were suspended in 10 µL of a solution containing 10 % acetonitrile, 0.1 % formic acid, 1 mg/mL bovine serum albumin. The washed beads were then suspended with 30 µL of 30% isopropanol,100 mM ammonium bicarbonate, and digested at 37 °C for 30 min after adding 100 µL of 0.01 mg/mL trypsin in 10 mM hydrochloric acid. The

liberated peptides were then removed from the beads using a magnet and analysed using LC-MS/MS.

Peptides were analysed by liquid chromatography-tandem mass spectrometry in the multiple reaction monitoring acquisition mode using an UHPLC I-Class Chromatography system coupled to a Xevo TQ-S triple quadrupole tandem mass spectrometer (Waters, MA, USA). Peptides were eluted from an Acquity UPLC HSS T3 1.8µm (C18, 2.1x50 mm, pore size 100 Å) analytical column (Waters) at 45 °C using 0.1 % formic acid, 2 % dimethylsulfoxide in LC-MS grade water as mobile phase A and 0.1 % formic acid, 2 % dimethylsulfoxide in LC-MS grade methanol as mobile phase B.

The liquid chromatography and mass spectrometry conditions are detailed in Supplementary Table 2, 3 and 4.

**Supplementary Table 2. PTH Liquid chromatography conditions**	
Mobile phase	Phase A: 0.1 % formic acid, 2 % dimethylsulfoxide in water<br>0.1 % formic acid, 2 % dimethylsulfoxide in methanol
Column	Acquity UPLC HSS T3 1.8µm (C18, 2.1x50 mm, pore size 100 Å)
Temperature	45±5 °C
Flow rate	0.3 mL/min
Injection volume	20 µL
Gradient	0-0.5 min: 2% B at 0.3 mL/min<br>7.5: 98% B at 0.3 mL/min<br>7.6: 98% B at 0.6 mL/min<br>8.6: 2% B at 0.6 mL/min<br>9.9: 2% at 0.3 mL/min

**Supplementary Table 3. PTH Mass spectrometry conditions**	
Source polarity	ESI+
Capillary voltage	3.25 kV
Source Offset voltage	50 V

Desolvation Temp	600 °C
Desolvation Gas Flow	1000 L/h
Cone Gas Flow	150 L/h

Supplementary Table 4. PTH Multiple reaction monitoring conditions

Peptide sequences	Q1 (m/z)	Q3 (m/z)	Cone (V)	Collision (V)	Ion type
HLNSMER.2	443.7136	218.1047	35	15	y3
HLNSMER.2	443.7136	261.6207	35	15	y4
HLNSMER.2	443.7136	318.6421	35	15	y5
HLNSMER.3	296.1448	218.1047	35	9	y3
HLNSMER.3	296.1448	261.6207	35	9	y4
HLNSMER.3	296.1448	318.6421	35	9	y5
HLNSMER.3	296.1448	435.202	35	9	y3

HLNSMER.3	296.1448	522.2341	35	9	y4
HLNSMER.3	296.1448	636.277	35	9	y5
HLNSM(+15.994915)ER.2	451.7111	226.1021	35	16	y3
HLNSM(+15.994915)ER.2	451.7111	269.6181	35	16	y4
HLNSM(+15.994915)ER.2	451.7111	326.6396	35	16	y5
HLNSM(+15.994915)ER.2	451.7111	451.1969	35	16	y3
HLNSM(+15.994915)ER.2	451.7111	538.229	35	16	y4
HLNSM(+15.994915)ER.2	451.7111	652.2719	35	16	y5
HLNSM(+15.994915)ER.3	301.4765	226.1021	35	10	y3
HLNSM(+15.994915)ER.3	301.4765	269.6181	35	10	y4
HLNSM(+15.994915)ER.3	301.4765	326.6396	35	10	y5
HLNSM(+15.994915)ER.3	301.4765	451.1969	35	10	y3

HLNSM(+15.994915)ER.3	301.4765	538.229	35	10	y4
ADVNVLTK.2	430.2478	574.3559	35	15	y5
ADVNVLTK.2	430.2478	673.4243	35	15	y6
ADVNVLTK.3	287.1676	181.1259	35	9	y3
ADVNVLTK.3	287.1676	230.6601	35	9	y4
ADVNVLTK.3	287.1676	361.2445	35	9	y3
ADVNVLTK.3	287.1676	460.313	35	9	y4
SLGEADK.2	360.1821	167.0921	35	12	y3
SLGEADK.2	360.1821	231.6134	35	12	y4
SLGEADK.2	360.1821	260.1241	35	12	y5
SLGEADK.2	360.1821	333.1769	35	12	y3
SLGEADK.2	360.1821	462.2195	35	12	y4

SLGEADK.2	360.1821	519.2409	35	12	y5
SLGEADK.3	240.4572	167.0921	35	7	y3
SLGEADK.3	240.4572	260.1241	35	7	y5
SLGEADK.3	240.4572	333.1769	35	7	y3
SLGEADK.3	240.4572	462.2195	35	7	y4
VEWLR.2	351.7003	229.1183	35	12	b2
VEWLR.2	351.7003	474.2823	35	12	y3
EDNVLVESHEK.2	649.8148	629.2889	35	23	y5
EDNVLVESHEK.2	649.8148	728.3573	35	23	y6
EDNVLVESHEK.2	649.8148	841.4414	35	23	y7
EDNVLVESHEK.3	433.5456	315.1481	35	14	y5
EDNVLVESHEK.3	433.5456	364.6823	35	14	y6

EDNVLVESHEK.3	433.5456	421.2243	35	14	y7
EDNVLVESHEK.3	433.5456	629.2889	35	14	y5
DAGSQRPR.2	443.7281	322.1854	35	15	y5
DAGSQRPR.2	443.7281	643.3634	35	15	y5
DAGSQRPR.2	443.7281	700.3849	35	15	y6
DAGSQRPR.3	296.1545	214.6401	35	9	y3
DAGSQRPR.3	296.1545	278.6693	35	9	y4
DAGSQRPR.3	296.1545	322.1854	35	9	y5
DAGSQRPR.3	296.1545	350.6961	35	9	y6
DAGSQRPR.3	296.1545	428.2728	35	9	y3
DAGSQRPR.3	296.1545	556.3314	35	9	y4
DAGSQRPR.3	296.1545	643.3634	35	9	y5

DAGSQRPR.3	296.1545	700.3849	35	9	y6
SVSEIQLMHNLGK.2	728.3849	527.2973	35	26	y9
SVSEIQLMHNLGK.2	728.3849	568.3202	35	26	y5
SVSEIQLMHNLGK.2	728.3849	635.3346	35	26	y11
SVSEIQLMHNLGK.2	728.3849	699.3607	35	26	y6
SVSEIQLMHNLGK.2	728.3849	812.4447	35	26	y7
SVSEIQLMHNLGK.2	728.3849	940.5033	35	26	y8
SVSEIQLMHNLGK.2	728.3849	1053.587	35	26	y9
SVSEIQLMHNLGK.2	728.3849	1269.662	35	26	y11
SVSEIQLMHNLGK.3	485.9257	159.1128	35	16	y3
SVSEIQLMHNLGK.3	485.9257	431.2613	35	16	y4
SVSEIQLMHNLGK.3	485.9257	470.7553	35	16	y8

SVSEIQLMHNLGK.3	485.9257	527.2973	35	16	y9
SVSEIQLMHNLGK.3	485.9257	568.3202	35	16	y5
SVSEIQLMHNLGK.3	485.9257	591.8186	35	16	y10
SVSEIQLMHNLGK.3	485.9257	635.3346	35	16	y11
SVSEIQLMHNLGK.3	485.9257	699.3607	35	16	y6
SVSEIQLMHNLGK.3	485.9257	812.4447	35	16	y7
SVSEIQLMHNLGK.3	485.9257	940.5033	35	16	y8
SVSEIQLM(+15.994915)HNLGK.2	736.3823	159.1128	35	26	y3
SVSEIQLM(+15.994915)HNLGK.2	736.3823	317.2183	35	26	y3
SVSEIQLM(+15.994915)HNLGK.2	736.3823	431.2613	35	26	y4
SVSEIQLM(+15.994915)HNLGK.2	736.3823	535.2948	35	26	y9
SVSEIQLM(+15.994915)HNLGK.2	736.3823	568.3202	35	26	y5

SVSEIQLM(+15.994915)HNLGK.2	736.3823	643.3321	35	26	y11
SVSEIQLM(+15.994915)HNLGK.2	736.3823	715.3556	35	26	y6
SVSEIQLM(+15.994915)HNLGK.2	736.3823	828.4396	35	26	y7
SVSEIQLM(+15.994915)HNLGK.2	736.3823	956.4982	35	26	y8
SVSEIQLM(+15.994915)HNLGK.2	736.3823	1069.582	35	26	y9
SVSEIQLM(+15.994915)HNLGK.3	491.2573	159.1128	35	16	y3
SVSEIQLM(+15.994915)HNLGK.3	491.2573	643.3321	35	16	y11

Data treatment

Data processing was performed with Skyline Daily version 21.1.1.223. The peak area for each peptide was determined as the sum of the peak areas of all selected transitions. The recovery over blocked-beads (RE) in spiked buffer and in spiked plasma was estimated using Equations 1, and 2, respectively.

$$RE_{buffer} = \frac{Peak\ area\ Series\ D}{Peak\ area\ Series\ B} \quad (1)$$

$$RE_{plasma} = \frac{Peak\ area\ Series\ H}{Peak\ area\ Series\ B} \quad (2)$$

Affinity Enrichment of Glucagon and Analysis by LC-MS/MS

Bead Preparation

Minibinder Beads

GCG binder-conjugated beads were prepared by binding 1-10 mg of cysteine-containing protein binder at the C-terminus to 30 mg of BcMag™ long-arm iodoacetyl-activated Magnetic Beads (Bioclone). Following the binding step, the excess active groups on the beads were blocked by adding 1 mL of 50 mM L-Cysteine•HCl and incubating for 60 minutes. As a control for binder specificity, an unrelated *de novo* protein binder was used in place of the GCG binder for the binder-negative control beads.

Monoclonal Antibody Beads

Anti-glucagon monoclonal antibody bound beads were prepared by binding 5 µg of in-house monoclonal antibody per prepared sample to 7.5 µL of tosyl M-280 beads (Thermofisher) Post-binding, beads are blocked with a Tris 0.1% BSA solution[40]. Two in-house monoclonal antibodies are bound separately and are pooled together prior to use. For monoclonal antibody negative control beads bovine gamma globulin was used in place of monoclonal antibody.

Immuno-Affinity Enrichment

Enrichment of glucagon peptide was assayed in triplicate for four different bead types: anti-glucagon monoclonal antibody tosyl beads, negative control tosyl beads, minibinder beads and negative control minibinder beads. PBS-CHAPS 0.1% was spiked with 62.5 pM exogenous glucagon, HSQGTFTSDYSKYLDSRRAQDFVQWLMNT (Anaspec) and 500 µL was added to 10 µL prepared monoclonal antibody beads and 50 µL of prepared minibinder beads. After 45 minutes of agitation at 1400 rpm at room temperature, the paramagnetic beads were washed 4 times 200 µL of PBS-CHAPS 0.1%. Samples were eluted for 8 minutes at 1400 rpm with 50 µL elution solvent (20% acetic acid 10% dimethylsulfoxide 10% acetonitrile 0.01% BSA in water) that was spiked with stable isotope labelled internal standard (New England Peptide). Elution solvent was also spiked with exogenous glucagon to mimic the peptide levels obtained if 100% of the peptide was captured by the antibody or minibinder ligands.

To prepare the beads for reuse after the elution step beads were resuspended in PBS-CHAPS 0.1% and pooled back together. On a subsequent day the beads were washed three times with elution solvent. Then they were washed three times in PBS-CHAPS 0.1%. Minibinder beads fresh, minibinder beads reused, mAb beads fresh and mAb beads reused were each tested in triplicate according to the same protocol listed above for the initial comparison.

LC-MS/MS Analysis

Samples were transferred to glass total recovery vials (Waters) and run on a Waters Acquity UPLC I-Class system with a flow through needle and coupled with a Waters TQ-S tandem mass spectrometer running in multiple reaction monitoring (MRM) mode. Liquid chromatography, mass spectrometer and MRM methods are summarised in Supplementary Table 5, 6 and 7, respectively. Enriched samples were injected in singlicate and the 100% recovery sample was injected in quintuplicate.

Supplementary Table 5. Glucagon Liquid Chromatography Conditions

Mobile Phase	A: 0.1% formic acid and 2% dimethylsulfoxide in water	
	B: 0.1% formic acid and 2% dimethylsulfoxide in methanol	
Column	Acquity UPLC HSS T3 1.8μm (C18, 2.1x50 mm, pore size 100 Å)	
Temperature	45±5 °C	
Injection Volume	35μL	
Gradient	0-0.5min	30% B at 0.3 mL/min
	7.0 min	5% B at 0.3mL/min
	7.1 min	95% B at 0.6 mL/min
	8.1min	30% B at 0.6 mL/min
	9.4 min	30% B at 0.3 mL/min

**Supplementary Table 6. Glucagon Mass Spectrometry Conditions**	
Source Polarity	ESI+
Capillary Voltage	1.4 kVA
Cone Offset	60 V
Desolvation Temperature	600 ℃
Desolvation Gas Flow	1000 L / hour
Cone Gas Flow	150 L / hour
Cone	35 V
Collision	24 V

**Supplementary Table 7. Glucagon Multiple Reaction Monitoring Conditions**			
Peptide Sequence	Q1 (m/z)	Q2 (m/z)	Ion Type
HSQGTFTSDYSKYLDSRRAQDFVQWLMNT	871.70	1002.74	b25
		1040.46	b26

		1084.19	b27
HSQGTF^TSDYSKYLDSRRAQDF^VQWLMNT	876.17	1008.69	b25 (IS)
		1046.41	b26 (IS)
F^ = 13C(+9)		1090.14	b27 (IS)

Chromatograms were analysed in Skyline Daily version 23.0.9.239[67,68]. Integrated peak areas were exported to Excel for further analysis. Results were calculated per these three equations. The results summary is shown in figure 4d (right) and representative chromatograms found in supplemental figure S17b.

$$Peak\ Area\ Ratio = \frac{sum\ Peak\ Area\ (b25 + b26 + b27)}{sum\ Internal\ Standard\ Peak\ area\ (b25(IS) + b26\ (IS) + b27\ (IS))}$$

$$Percent\ Recovery\ = \frac{Peak\ Area\ Ratio\ of\ Sample}{Average\ Peak\ Area\ Ratio\ of\ 100\%\ Recovery\ sample\ (n = 5)}$$

$$Normalized\ Percent\ Recovery\ = \frac{Percent\ Recovery\ of\ Sample}{Average\ Percent\ Recovery\ of\ mAb\ sample\ (n = 3)}$$

References

59. Virtanen, P. *et al.* SciPy 1.0: fundamental algorithms for scientific computing in Python. *Nat. Methods* **17**, 261–272 (2020).

60. Xia, Y. *et al.* T5 exonuclease-dependent assembly offers a low-cost method for efficient cloning and site-directed mutagenesis. *Nucleic Acids Res.* **47**, e15 (2019).

61. Kabsch, W. XDS. *Acta Crystallogr. D Biol. Crystallogr.* **66**, 125–132 (2010).

62. Winn, M. D. *et al.* Overview of the CCP4 suite and current developments. *Acta Crystallogr. D Biol. Crystallogr.* **67**, 235–242 (2011).

63. McCoy, A. J. *et al.* Phaser crystallographic software. *J. Appl. Crystallogr.* **40**, 658–674

(2007).

64. Adams, P. D. *et al.* PHENIX: a comprehensive Python-based system for macromolecular structure solution. *Acta Crystallogr. D Biol. Crystallogr.* **66**, 213–221 (2010).

65. Emsley, P. & Cowtan, K. Coot: model-building tools for molecular graphics. *Acta Crystallogr. D Biol. Crystallogr.* **60**, 2126–2132 (2004).

66. Williams, C. J. *et al.* MolProbity: More and better reference data for improved all-atom structure validation. *Protein Sci.* **27**, 293–315 (2018).

67. MacLean, B. *et al.* Skyline: an open source document editor for creating and analyzing targeted proteomics experiments. *Bioinformatics* **26**, 966–968 (2010).

68. Pino, L. K. *et al.* The Skyline ecosystem: Informatics for quantitative mass spectrometry proteomics. *Mass Spectrom. Rev.* **39**, 229–244 (2020).

69. Wojdyla, J. A., Fleishman, S. J., Baker, D. & Kleanthous, C. Structure of the ultra-high-affinity colicin E2 DNase--Im2 complex. *J. Mol. Biol.* **417**, 79–94 (2012).

70. Crooks, G. E., Hon, G., Chandonia, J.-M. & Brenner, S. E. WebLogo: a sequence logo generator. *Genome Res.* **14**, 1188–1190 (2004).

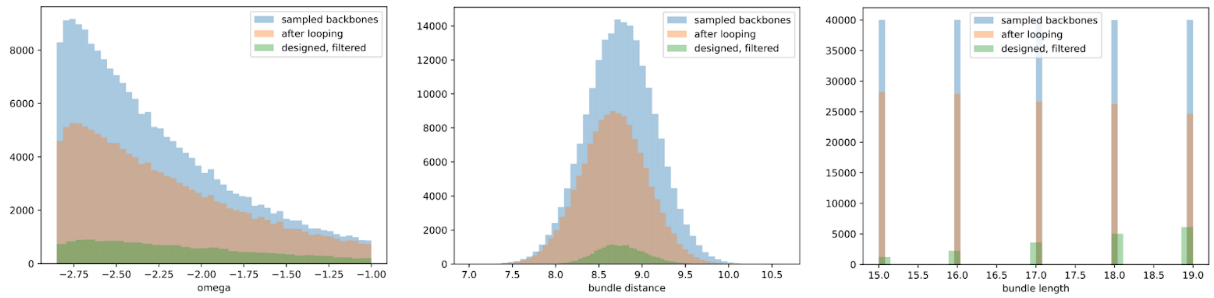

Figure S1: Sampled parameter distributions for parametric groove scaffolds. Left: Distributions of supercoiling (omega). Middle: Average distance between neighbouring helices (bundle distance). Middle: Length of helices in scaffold bundle (bundle length) for sampled parametric groove scaffolds. Distributions are depicted for each step of the design process: initial parametrically sampled helix arrangements, scaffold backbones after loop closure, and final designed scaffolds after filtering.

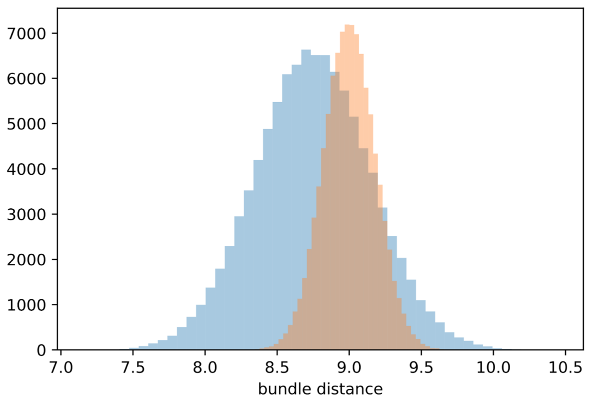

Figure S2: Sampling strategy for average helical neighbour distance. First, a global average helix neighbour distance (bundle distance) was randomly selected for each sampled scaffold from a Gaussian distribution informed by native protein helical bundle geometries (blue). Then, the distance of each helix from its neighbouring helices for a single scaffold was independently randomly sampled from a tighter normal distribution (orange) centred around the selected global average helix neighbour distance (for this demonstrative example 9.0).

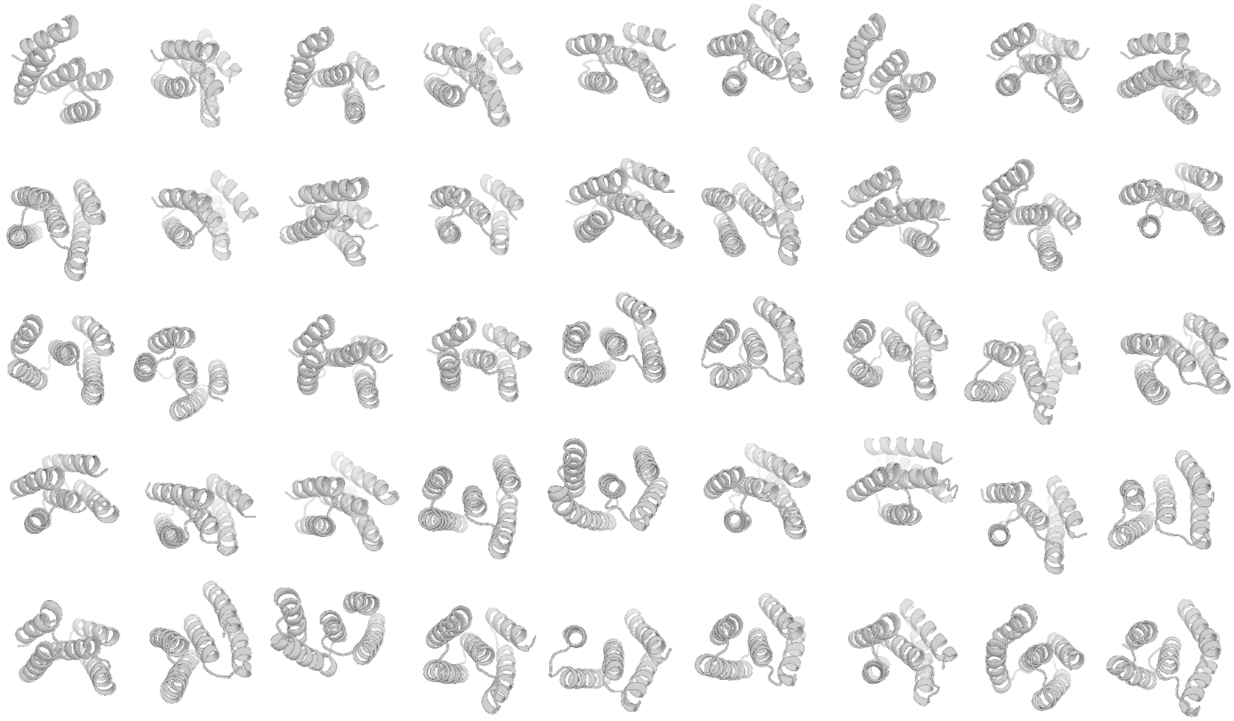

Figure S3: Parametric groove scaffold library: 45 scaffolds from the library of 18 thousand parametric groove scaffolds, demonstrating a range of supercoiling and helix distances to accommodate a range of helical peptide targets.

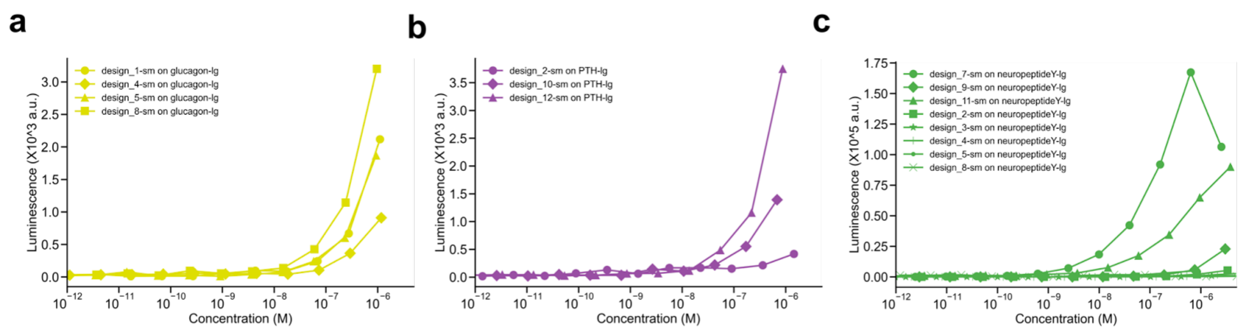

Figure S4: NanoBiT binding curves for initial parametric binder hits show weak affinities. (a) Glucagon (yellow), **(b)** PTH (purple), and **(c)** NPY (green) titration binding curves as measured through a nanoBiT split luciferase assay show weak binding, in most cases indistinguishable from the background signal of the two split luciferase halves binding (190 μM).

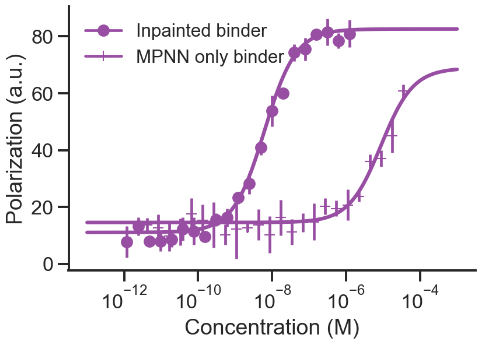

Figure S5. Comparison of binding affinities between the PTH Inpainted binder and the parametrically redesigned binder using ProteinMPNN only. Structural extension with RF_{joint} Inpainting and ProteinMNN sequence redesign resulted in a significant increase in the binding affinity of the parametrically designed PTH binder. Conversely, ProteinMNN redesign performed solely on the original parametrically designed binder did not lead to an increase in affinity.

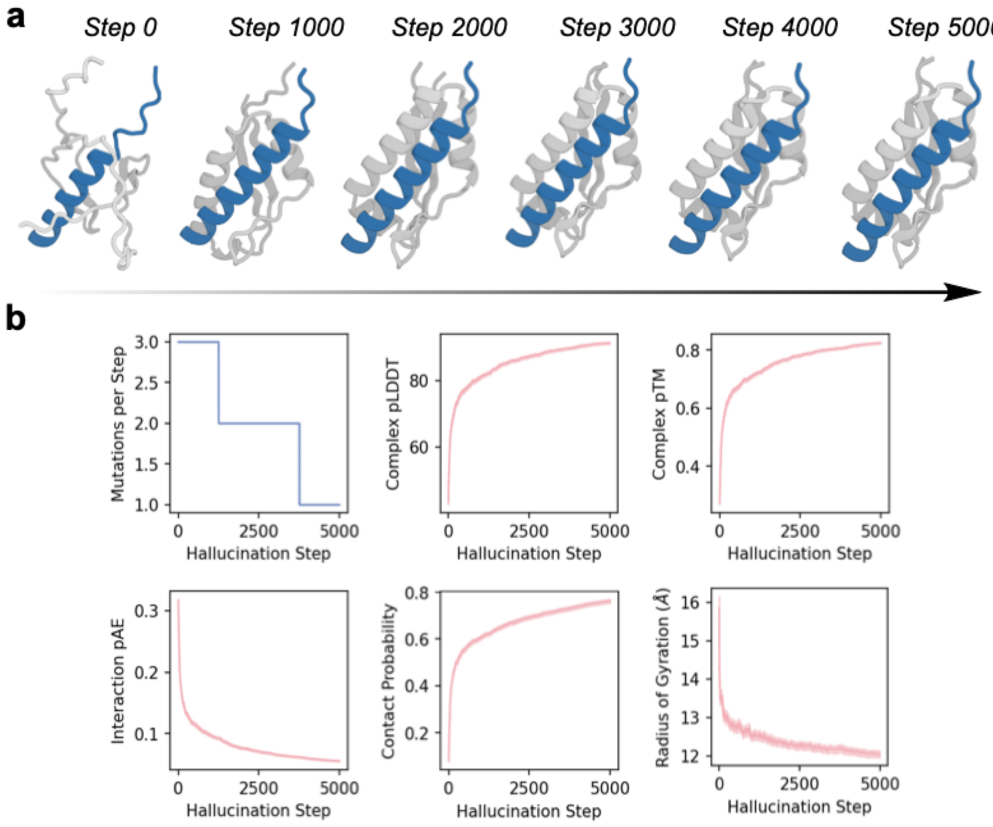

Figure S6: Hallucinating Bid Binders with AlphaFold2: (a) Example hallucination trajectory generating 70 amino acid binders to the peptide Bid (blue). Initially, AlphaFold2 predicts an unstructured "binder", but over 5000 steps, a binder is built up around the peptide. Crucially, no template structure is provided for the Bid peptide, allowing AF2 to predict its structure throughout. Note the predicted elongation of the helical structure in the peptide (blue, top) over the hallucination trajectory. **(b)** Hallucination trajectories approximately converge after 5000 steps. Left to right, top to bottom: The mutation rate at each step is decayed throughout the trajectory (1250 x 3 steps, 2500 x 2 steps, 1250 x 1 step). More mutations initially helps speed up hallucination, while a lower rate later on allows more gradual refinement. The AF2 confidence (pLDDT, pTM) in the bound structure increases throughout trajectories, while the interaction pAE decreases. The contact probability also trends to convergence over the trajectories, while the proteins typically become more compact (radius of gyration). N=96 trajectories.

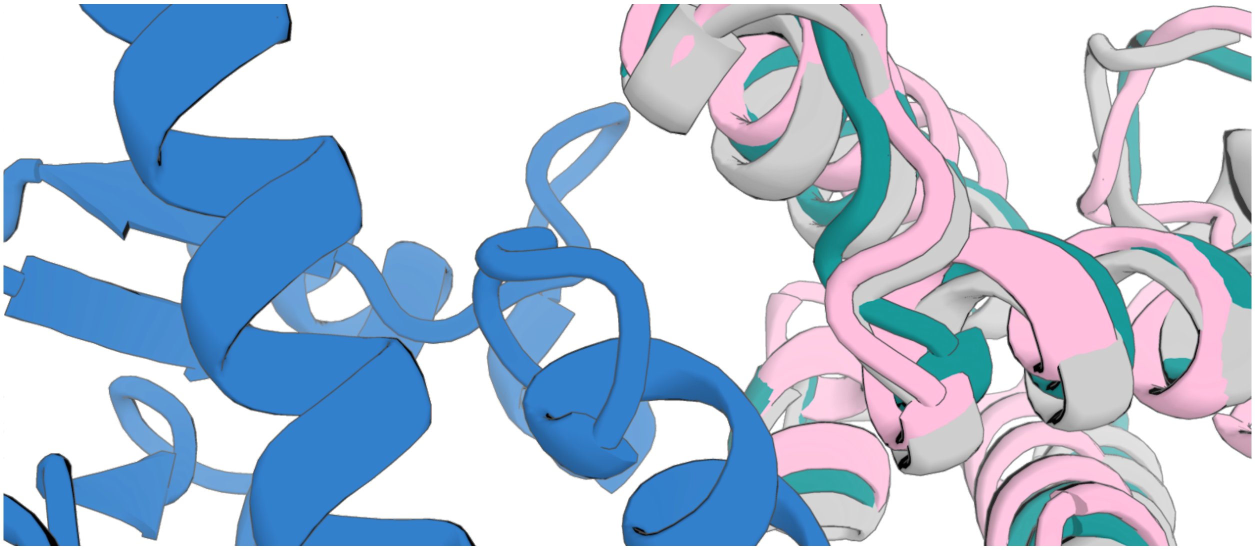

Figure S7. Specificity redesign of a native immunity protein. Colicin-E2 immunity protein[69] (teal, PDB ID: 3U43) was superimposed on the PDB structure (PDB ID: 1EMV) of the native Immunity protein E9[35] (grey) - colicin E9 structure (blue). The superimposed structure serves as the starting point for conducting 100 designs using partial diffusion (60 steps, noise=1) and ProteinMPNN (T=0.1) vs ProteinMPNN alone (T=0.1). The latter resulted in only one structure being predicted as a complex using AlphaFold (interaction pAE < 20) while the former had 7 structures predicted to form a complex. The AlphaFold model of the top-ranked design is shown in pink. Notably, the model reveals a subtle movement in the partially diffused structure of the "α1–α2 motif", a major specificity determinant, which accurately recapitulates the native interaction observed in the colicin E9 complex[35]. The native complex was not part of the training set used to train the diffusion model.

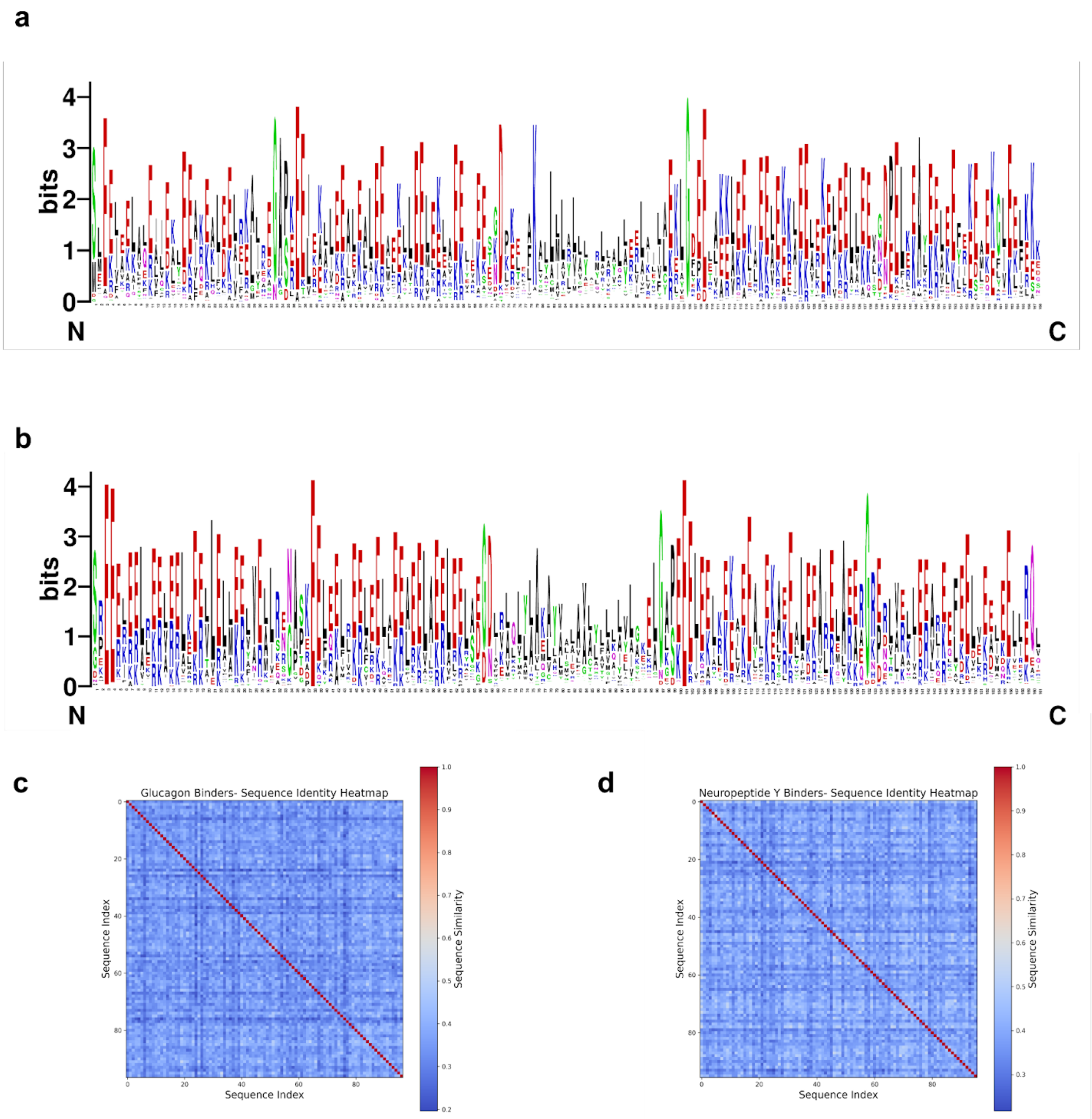

Figure S8. Sequences designed after partial diffusion are highly diverse. (a) Sequence logo for the designed binders generated through partial diffusion for GCG and **(b)** NPY. In both cases, the computationally designed binders show rich diversity in sequence space as visualised in their sequence logos[70]. **(c)** All-by-all heatmap of sequence identity for the designs tested against GCG and **(d)** NPY further demonstrate sequence diversity of the design proteins.

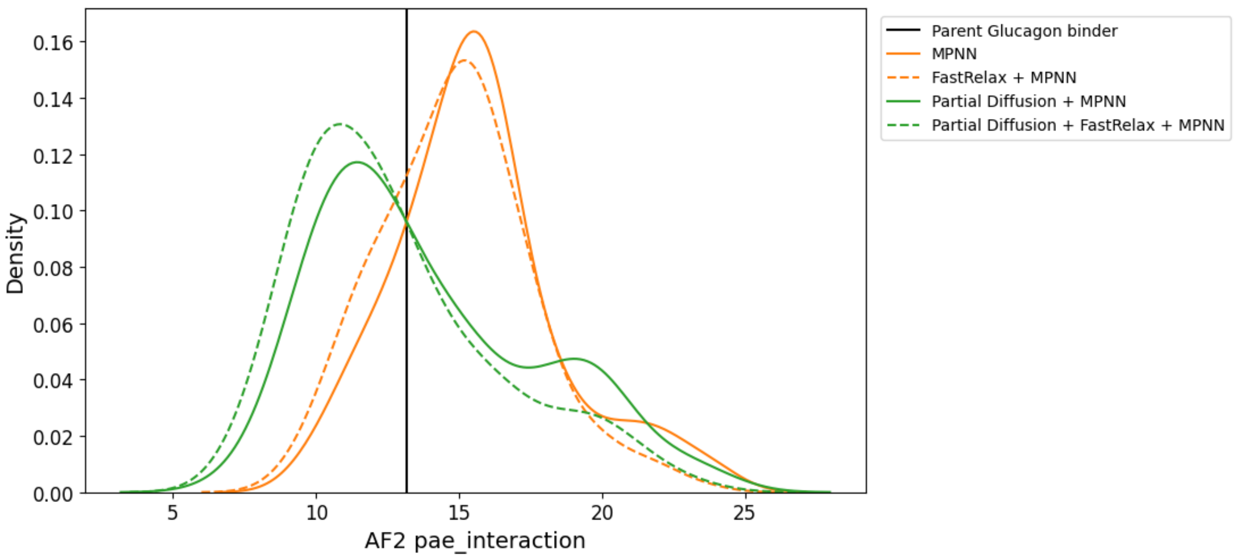

Figure S9. Partial Diffusion is necessary for significant improvement in binding metrics. An AlphaFold2 predicted model of a weak binder to GCG (K_d ~ 200 nM) was used as a starting structure to improve in-silico binding metrics through different methods. Using this parent design, we ran 1) ProteinMPNN (T=0.1) alone, 2) ProteinMPNN (T=0.1) followed by FastRelax and a second round of ProteinMPNN (T=0.1), 3) Partial diffusion (40 steps) followed by ProteinMPNN (T=0.1), and 4) Partial diffusion (40 steps), followed by ProteinMPNN (T=0.1), FastRelax and a second round of ProteinMPNN (T=0.1). 200 sequences were generated using each method. Binding metrics based on AlphaFold2 clearly show that while ProteinMPNN and FastRelax are occasionally able to improve the parent design, the distribution is significantly improved when partial diffusion is used to generate the backbone ensemble first. FastRelax offers a further marginal improvement.

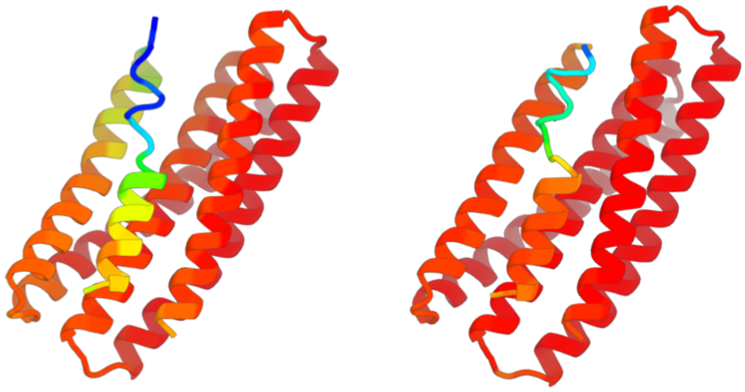

Figure S10. AF2 predictions of the Inpainted and partially diffused GCG binders. The Inpainted (left) and partially diffused (right) binders to GCG are coloured (spectrum blue to red, low to high) by pLDDT.

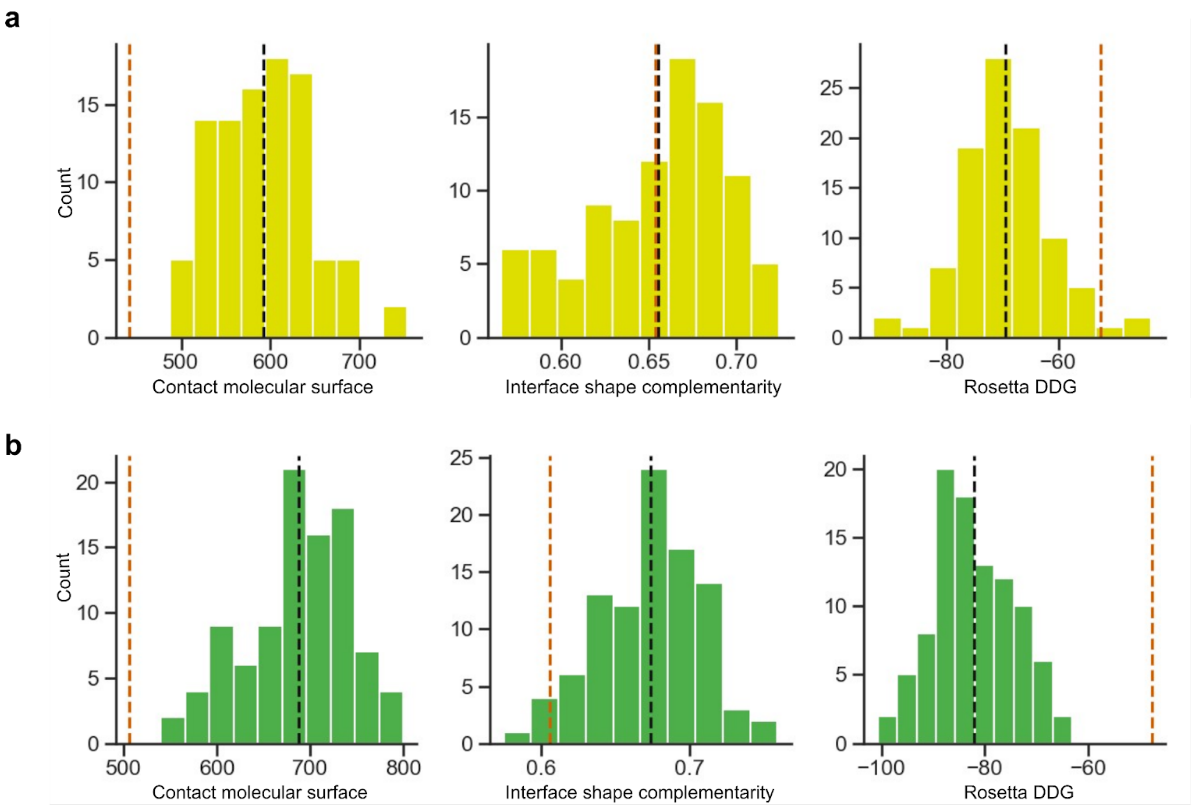

Figure S11. Binding metrics for partially diffused binders. (a) Computational metrics for 96 tested partially diffused glucagon binders showed significant improvement in contact molecular surface (a measure of interface size and quality) and Rosetta ddG (a measure of interface predicted energy) over the starting design (vertical red lines). Distribution means are shown in black. **(b)** Computational metrics for 96 ordered partially diffused NPY binders showed significant improvement in contact molecular surface, Rosetta ddG, and interface shape complementarity (a measure of interface quality) over the starting design (vertical red lines). Means are shown in black.

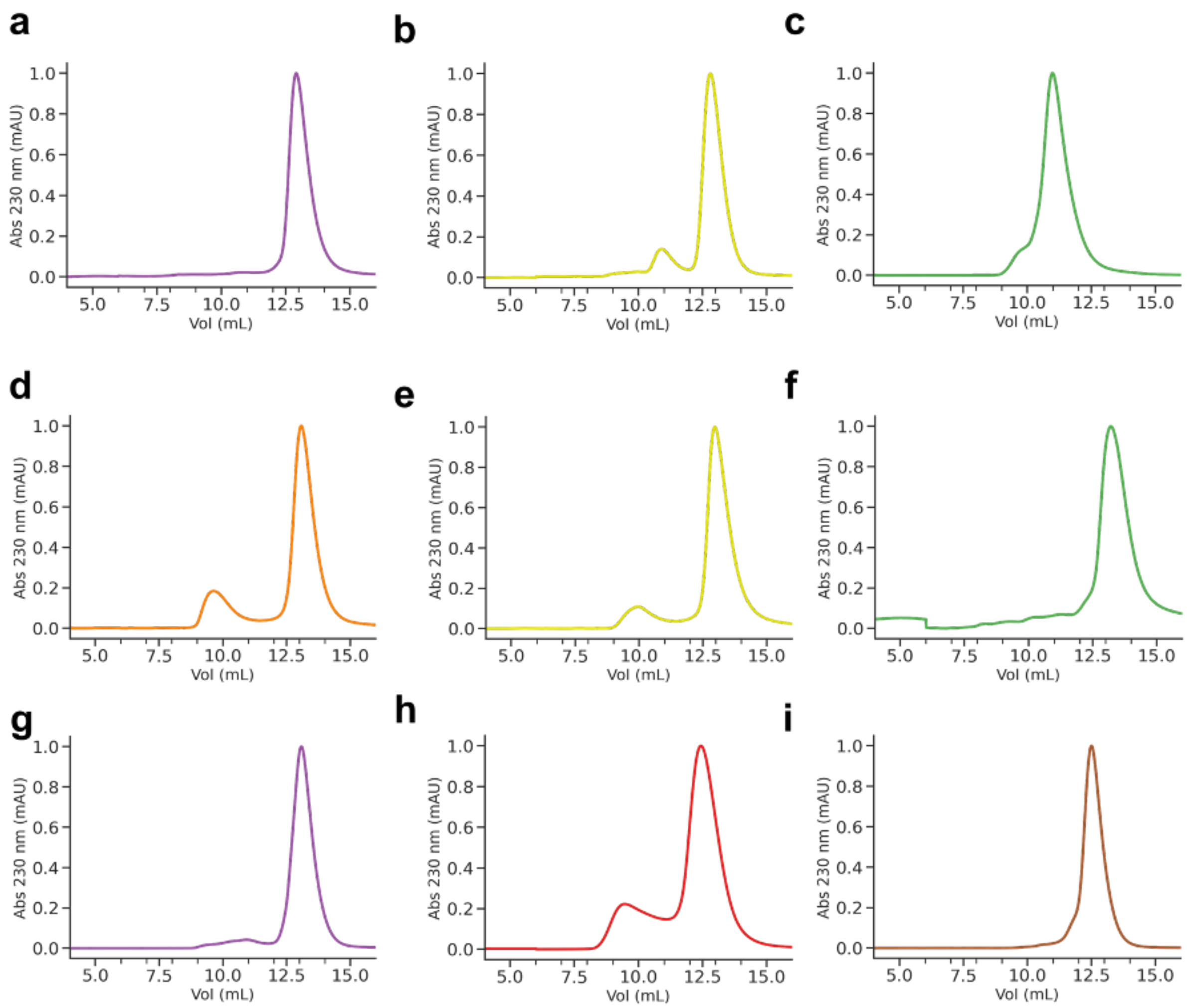

Figure S12. SEC traces of peptide binders. (a) SEC reinjection of the Inpainted PTH binder. **(b)** SEC reinjection of the Inpainted GCG binder. **(c)** SEC reinjection of the Inpainted NPY binder. **(d)** SEC reinjection of the threaded SCT binder. **(e)** SEC reinjection of the partially diffused GCG binder. **(f)** SEC reinjection of the partially diffused NPY binder. **(g)** SEC reinjection of the fully diffused PTH binder. **(h)** Initial SEC of the fully diffused Bim binder. **(i)** SEC reinjection of the flexibly diffused PYY binder. All reinjections were of the most abundant monodisperse peak and were performed 24 hours after initial SEC.

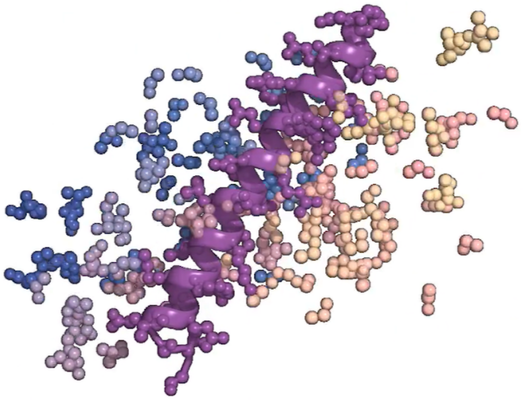

Supplementary Video 1. A video of the diffusion trajectory for the fully diffused PTH binder can be seen at https://www.bakerlab.org/wp-content/uploads/2022/11/diffusion_animation_PTHbinder_v6.mp4

Computational method	Targeted peptides
Parametric design	GCG, PTH and NPY
Hallucination	Bid
RF_{joint} Inpainting	Parametrically designed binders for GCG, PTH and NPY
Threading design	PTH, NPY, GCG, SCT, GIP, GLP1, GLP2
Partial diffusion	Inpainted GCG and NPY binders
Unconditional diffusion	PTH and Bim
RFdiffusion	PYY, NPY, GCG, Puma and PTH

Supplementary Table 8. Targeted peptides by each of the presented computational design approaches.

	GCG Binder	NPY Binder	PTH Binder	Bim Binder
**RMSD AF2 vs Design**	0.62 Å	0.61 Å	0.78 Å	0.80 Å
**AF2 interaction pAE**	9.25	8.29	4.40	4.50
**AF2 pLDDT for binder**	95.52	93.41	94.3	96.6

Supplementary Table 9. AlphaFold2 metrics for partially and fully diffused binders.
