# Supplementary figures and images for: De novo design of high-affinity binders of bioactive helical peptides
Source: Nature. 2023 Dec 18;626(7998):435–42. doi: 10.1038/s41586-023-06953-1 (PMC10849960; doi:10.1038/s41586-023-06953-1)

|              | 29 |   |   |   |   | 361 |   |   |   |   |
|--------------|----|---|---|---|---|-----|---|---|---|---|
| Strep. beads | -  | + | + | + | + | -   | + | + | + | + |
| Bid-BTN      | -  | + | + | - | - | -   | + | + | - | - |
| L            |    | U | B | U | B |     | U | B | U | B |

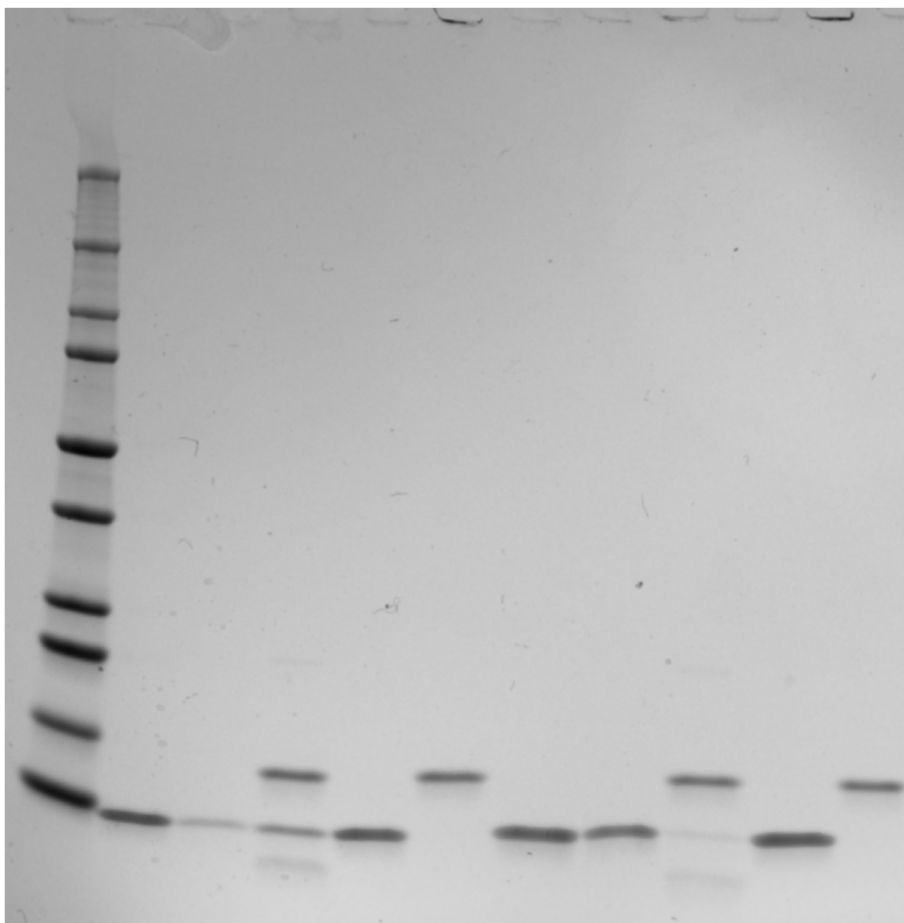

|              | 410 |   |   |   |   | 411 |   |   |   |   |
|--------------|-----|---|---|---|---|-----|---|---|---|---|
| Strep. beads | -   | + | + | + | + | -   | + | + | + | + |
| Bid-BTN      | -   | + | + | - | - | -   | + | + | - | - |
| L            |     | U | B | U | B |     | U | B | U | B |

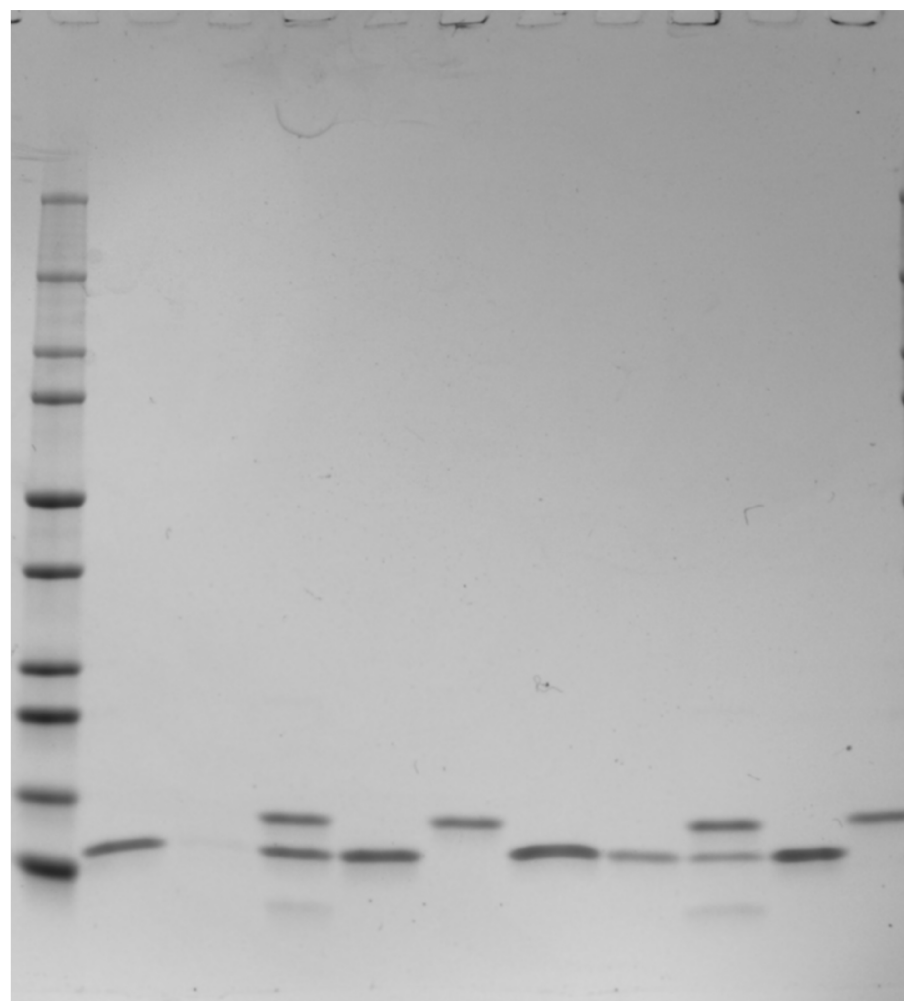

Strept.  
Binder  
Bid

Supplement: Supplementary file 3 — Uncropped blots. [file 41586_2023_6953_MOESM3_ESM.pdf]
